# The role of age in ocular toxoplasmosis: clinical signs of immunosenescence and inflammaging

**DOI:** 10.3389/fmed.2024.1311145

**Published:** 2024-03-05

**Authors:** Armin Taghavi Eraghi, Justus G. Garweg, Uwe Pleyer

**Affiliations:** ^1^Augenklinik, Charité Campus Virchow Klinikum, Universitätsmedizin Berlin, Berlin, Germany; ^2^Swiss Eye Institute, Rotkreuz, Zug, Switzerland; ^3^Berner Augenklinik, Bern, Switzerland; ^4^Klinik und Poliklinik für Augenheilkunde, Inselspital, Universität Bern, Bern, Switzerland; ^5^Berlin Institute of Health, Berlin, Germany

**Keywords:** age, antibody index, immune response, ocular toxoplasmosis, *Toxoplasma gondii*, uveitis, immunosenescence, inflammaging

## Abstract

**Purpose:**

This study aimed to investigate the association between age, immune response, and clinical presentation of ocular toxoplasmosis (OT).

**Design:**

This was a monocentric, retrospective, observational cohort study.

**Methods:**

A review of the medical records of patients with active OT at the Uveitis Center, Charité Universitätsmedizin, was conducted. Baseline parameters included age at presentation, visual acuity, intraocular pressure (IOP), size and location of active lesions, inflammatory activity, antibody index (AI), and complications of intraocular inflammation. The data were presented as the mean ± standard deviation (SD). The level of significance was set at a *p*-value of <0.05.

**Results:**

Between 1998 and 2019, 290 patients with active OT were diagnosed at our tertiary reference center. The mean age of the participants was 37.7 ± 17.1 years, 53.8% of them were female individuals, and 195 patients (70.9%) showed recurrent disease. Older age was associated with lower baseline visual acuity (*p* = 0.043), poor visual outcome (*p* = 0.019), increased inflammatory activity (*p* < 0.005), and larger retinal lesions (*p* < 0.005). Older patients presented a lower AI (<35 years: 45.1 ± 82.7, median: 12.1; ≥35 years: 18.6 ± 50.5, median: 5.8; *p* = 0.046), confirmed by a decrease in AI with increasing age (*R*^2^ = 0.045; *p* = 0.024). Finally, AI was correlated with lesion size (multiple linear regression analysis: *p* = 0.043). Macular involvement (24.3% of patients) was positively correlated with complications (macular/peripapillary edema and retinal detachment, *p* < 0.005) and poor visual outcome (*p* < 0.005) and was negatively correlated with inflammatory activity (*p* < 0.005).

**Conclusion:**

We found a strong and clinically relevant impact of age on the clinical presentation and course of OT. While an unspecific inflammatory response increased with age, the specific, local humoral immune response declined. These findings are well in line with the concept of immunosenescence and inflammaging in uveitis.

## GRAPHIC ABSTRACT

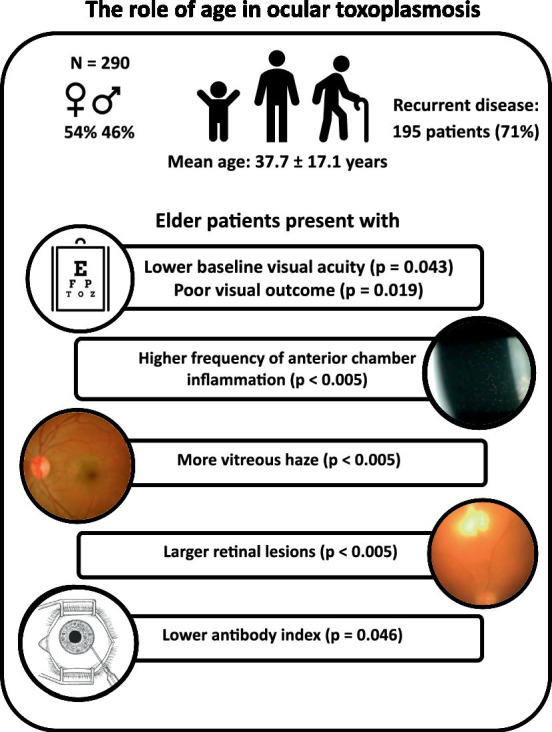

## 1 Introduction

The majority of infections in Europe with the protozoal parasite, *Toxoplasma gondii,* are related to the archetypical type 2 strains that have relatively low virulence (1, 2). These strains result in persistent (i.e., lifelong) infections as a consequence of cyst formation in virtually all tissues of the body, particularly the eye and the brain, given the neurotropism of this parasite (3). Virtually all warm-blooded hosts can develop a chronic infection, which explains the high evolutionary success of this global parasite (4). In human, the vast majority of infections remain asymptomatic throughout life, while congenital infections and infections in immunocompromised hosts may result in severe organ damage (5). Only a small portion of immunocompetent patients will experience organ damage, which typically affects the eye (4, 6).

Age and the individual’s immune response appear to be key factors influencing the clinical course and the risk of recurrent disease in chornic systemic toxoplasmosis (7–9). In contrast, the relevant impact of different parasite strain types is negligible, given the overwhelming dominance of infections with type 2 strains in the European population (10–13). It is hardly surprising that patient age has also been discussed as a potentially relevant factor for clinical manifestation and course in ocular toxoplasmosis (OT) (7, 12, 14–17). Based on current evidence and depending on the definition of outcomes, it seems that more severe courses of OT are present in the extreme age groups, i.e., either in congenital infections or at older ages. Age could possibly be linked to the route of infection (18), i.e., a higher incidence and severity of acquired OT in elderly persons (19); however, available data are inconsistent (20, 21), which may be partially explained by the limited sample size of the published cohorts. In addition, socioeconomic factors, the geographic region of a study, and the follow-up period may influence the outcomes (20, 22). Since the population continues to age globally and the infection rates remain generally high among older individuals (23), understanding the impact of age on the clinical presentation of OT is of increasing importance. Therefore, this retrospective cohort study aimed to investigate the potential role of patient age on the clinical presentation of OT.

## 2 Patients and methods

In this single-center, retrospective cohort study, we evaluated the medical records of 290 patients with active OT that presented between 1998 and 2019 in the Uveitis Clinic at the University Department of Ophthalmology, Charité Campus Virchow Klinikum, Berlin, Germany. The diagnosis was based on the discretion of the responsible physician (UP) and on clinical grounds and was supported by further analyses including serology and aqueous humor analysis.

All findings reported below were extracted from the patients’ medical records at the initial visit (baseline) and after “healing.” Healing was a sharp demarcation of the previously active retinochoroidal lesion with the formation of a pigmented chorioretinal scar and the subsidence of inflammation in the affected eye.

Beyond demographic parameters, patient age at the diagnosis of a new active lesion, as well as the patient’s geographic origin, gender, and immune status, were recorded. The following ophthalmic findings were recorded at baseline: unilateral or bilateral affection; number of active lesions, including lesion location and size; presence and location of preexisting chorioretinal scars; grading of inflammation according to the Standardization of Uveitis Nomenclature (SUN) criteria (24); intraocular pressure (IOP); and complications.

The size of active OT lesions was compared to the optic disc diameter (ODD) of the affected eye and categorized into four clusters (cluster 1: 0.1–1.4 ODD, cluster 2: 1.5–2.4 ODD, and so on). In the case of multiple active lesions, the largest focal lesion was considered. A total of 86 funduscopic images were available for the metric analysis using software FIJI-ImageJ Version 1 (25) and were included in the regression analysis. The most central lesion was used for the anatomical grading into macular, juxta/peripapillary, and peripheral retinal localization to compare between macular and extramacular, as well as central (macular and papillary) and peripheral lesions. IOP (mmHg) was quantified using a Goldmann applanation tonometer. Values between 10 and 22 mmHg were considered normal (IOP ≥22: elevated IOP). The presence of macular edema (ME), optic nerve head (ONH) involvement, and retinal detachment was registered as complications for the purpose of this study. If both eyes were affected, the eye with more severe inflammatory activity was included in this evaluation as having inflammatory activity.

### 2.1 Definitions

The baseline examination refers to the first examination during an active episode of OT in our institution, regardless of the duration of symptoms. We assumed a primary OT in the presence of a fresh OT lesion in the absence of old scars in either eye. A recurrence was correspondingly defined as an active lesion in the presence of a pigmented scar or a history of previously confirmed OT. Serological testing was not a prerequisite to support the diagnosis.

The age at diagnosis of primary OT was defined as that at the first manifestation of an active lesion in the absence of scars. In recurrent disease, the age at the first episode of OT was also recorded, if available. The age of patients with primary OT due to confirmed congenital toxoplasmosis was defined as 0 years. The duration of an active OT episode was quantified in weeks, from the initial presentation to the scarring of the lesion.

The majority of patients (264 out of 290 patients; 91.0%) received one of the two standard treatment regimens (clindamycin or cotrimoxazole) used during the study period for a duration of 4 to 6 weeks, along with systemic and topical corticosteroids at the discretion of the treating physician team (26–28). In 21 instances, clindamycin treatment had to be switched to cotrimoxazole due to side effects.

### 2.2 Statistical analysis

For statistical purposes, the cohort was metrically divided into two groups according to the median age of 34 years. Group 1 included 146 patients below 35 years, while group 2 included 144 patients aged 35 years or older. Age was additionally introduced as a continuous variable in the correlation analyses, along with best-corrected visual acuity (BCVA) at baseline and after therapy, the severity of inflammation, lesion size, the presence of complications (ME, ONH involvement, and retinal detachment), IOP, time to healing, lesion location, route of infection (congenital vs. acquired), primary vs. recurrent disease, and immune state. For statistical purposes, Snellen BCVA values were converted to the logarithm of the minimal angle of resolution (logMAR). Descriptive statistics were used to report the data from this non-comparative cohort study. According to the Shapiro–Wilk test, the data were not normally distributed. Due to the retrospective nature of data collection, data were variably missing. These missing data were not replaced.

Data are presented as the mean ± standard deviations (SDs), as the median, and as 25%–75% interquartile ranges (IQRs), if not otherwise indicated. A chi-square test was applied to compare independent distribution patterns within groups, a student’s *t*-test and a Mann–Whitney *U*-test to compare the means of two groups, analysis of variance (ANOVA) to compare the means of more than two groups, and a linear regression analysis to explore the possible associations between the dependent and independent variables. Additionally, multivariate logistic regression analyses were performed to identify the relationships between different individual factors simultaneously. A *p*-value of <0.05 was considered statistically significant.

## 3 Results

### 3.1 Age and gender distribution

During the study period (1998–2019), a total of 290 patients (290 eyes) presented with an episode of active OT. The age at presentation was 37.7 (±17.1; median 34; 8–86) years, which was similar for both men and women. Following the exclusion of patients with congenital disease, patients with primary OT were marginally older than patients with recurrent OT (Table 1).

**Table 1:** Demographic data.

	*N* (%)	Mean (years)	Standard deviation	Median	*p*-value
Cohort (total)	290 (100)	37.7	17.1	34	—
Men	134 (46.2)	38.4	17.1	35.5	0.44
Women	156 (53.8)	37.0	17.1	34	—
Congenital OT at presentation	27 (9.3)	29.2	14.3	30	0.008
Acquired and/or undetermined OT	263 (90.7)	38.5	17.1	35	—
Primary OT at presentation	80 (29.1)	38.2	19.3	32	0.87
Recurrent OT at presentation	195 (70.9)	36.3	15.5	34	—
Primary postnatally acquired (primary + anamnestic history of primary episode)	206 (71.0)	32.4	17.1	29	—
Unilateral presentation	229 (79.0)	37.8	16.3	34	0.6
Bilateral presentation	61 (21.0)	37.3	19.8	34	—

### 3.2 Association between visual acuity and age at presentation

**Figure 1:**
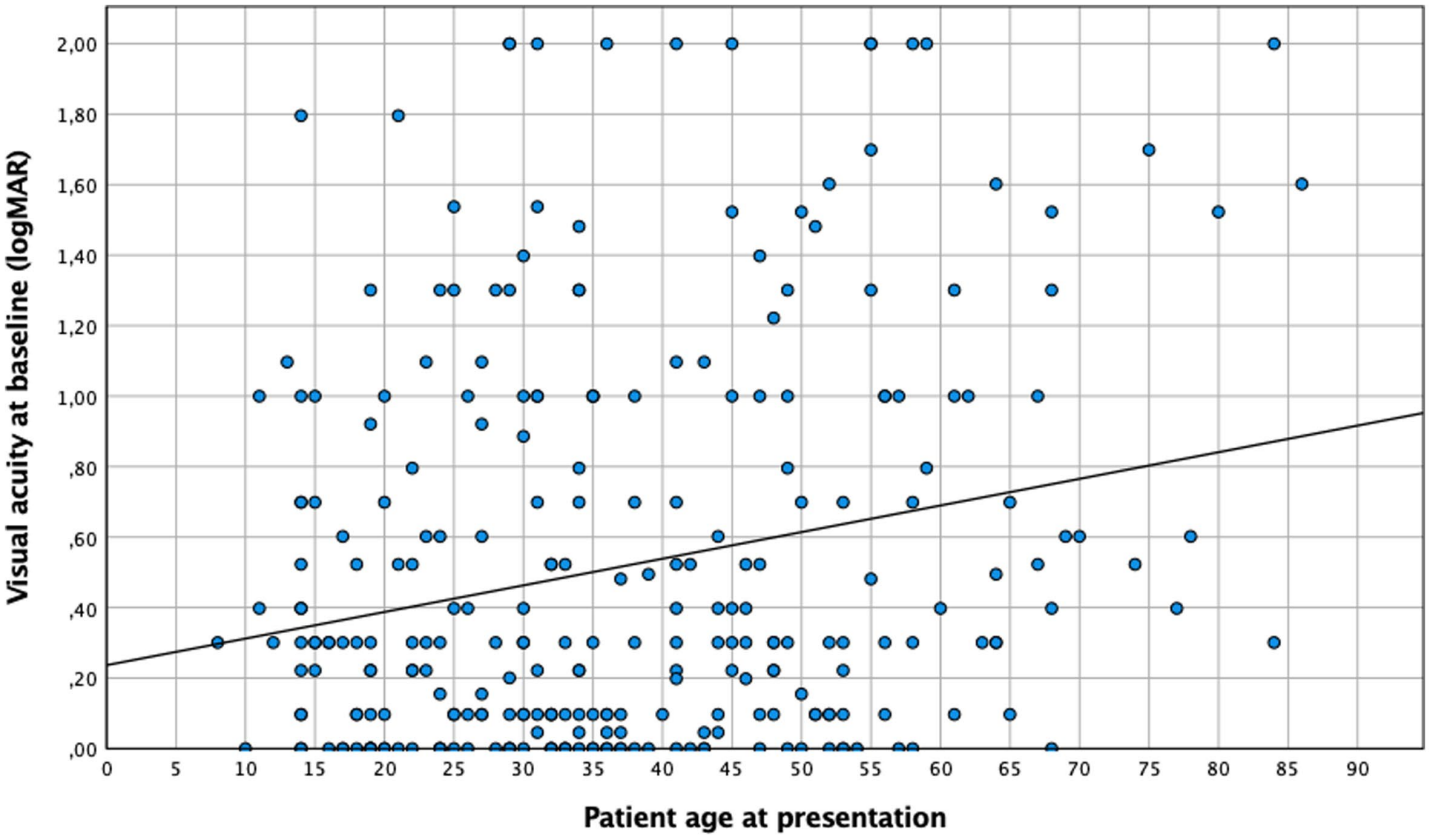
Visual acuity at diagnosis and patient age. Linear regression: *p* < 0.005, *n* = 275.

**Figure 2:**
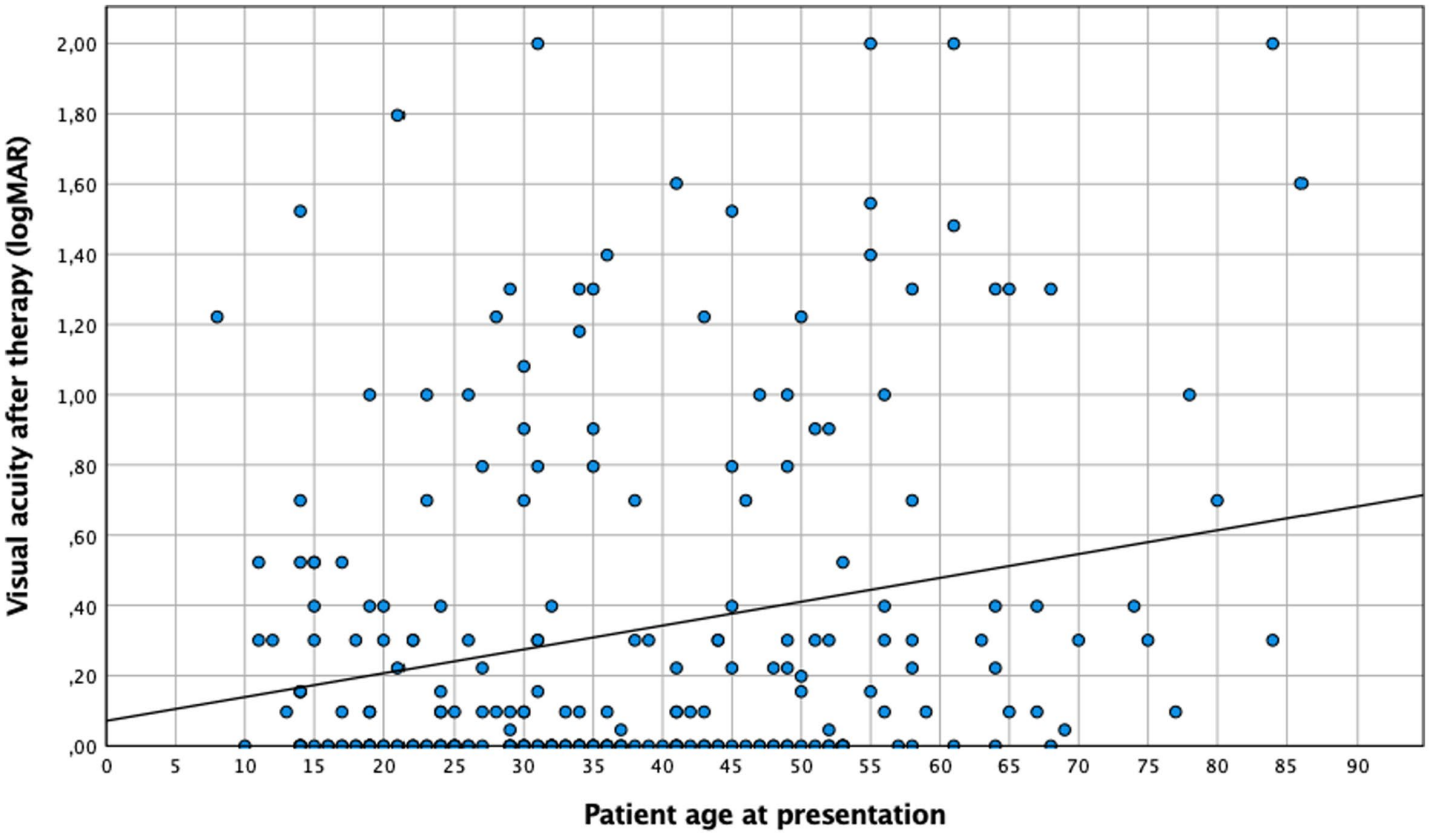
Visual acuity after therapy and patient age. Linear regression: *p* < 0.005, *n* = 240.

Patients over 35 years tended to present a lower baseline visual acuity than younger ones, both before (0.45 logMAR vs. 0.59 logMAR ≥35 years; *n* = 275, *p* = 0.043) and after therapy (0.25 logMAR vs. 0.4 logMAR ≥35 years; *n* = 240, *p* = 0.019). This finding was consistent if age was considered a continuous variable (Figures 1, 2). No difference in “change” in visual acuity before and after therapy (0.73 logMAR vs. 0.82 logMAR ≥35 years; *n* = 235, *p* = 0.2) was observed; however, to rule out a possible ceiling effect (BCVA of 1.0; logMAR = 0; 28 patients <35 years; and 20 patients ≥35 years), patients without vision loss at baseline and after treatment were excluded, and an association between BCVA and age became evident (change in visual acuity in patients <35 years was 0.62 logMAR compared to 0.74 logMAR in those ≥35 years; n = 187, *p* = 0.036).

### 3.3 Clinical presentation and age

**Figure 3:**
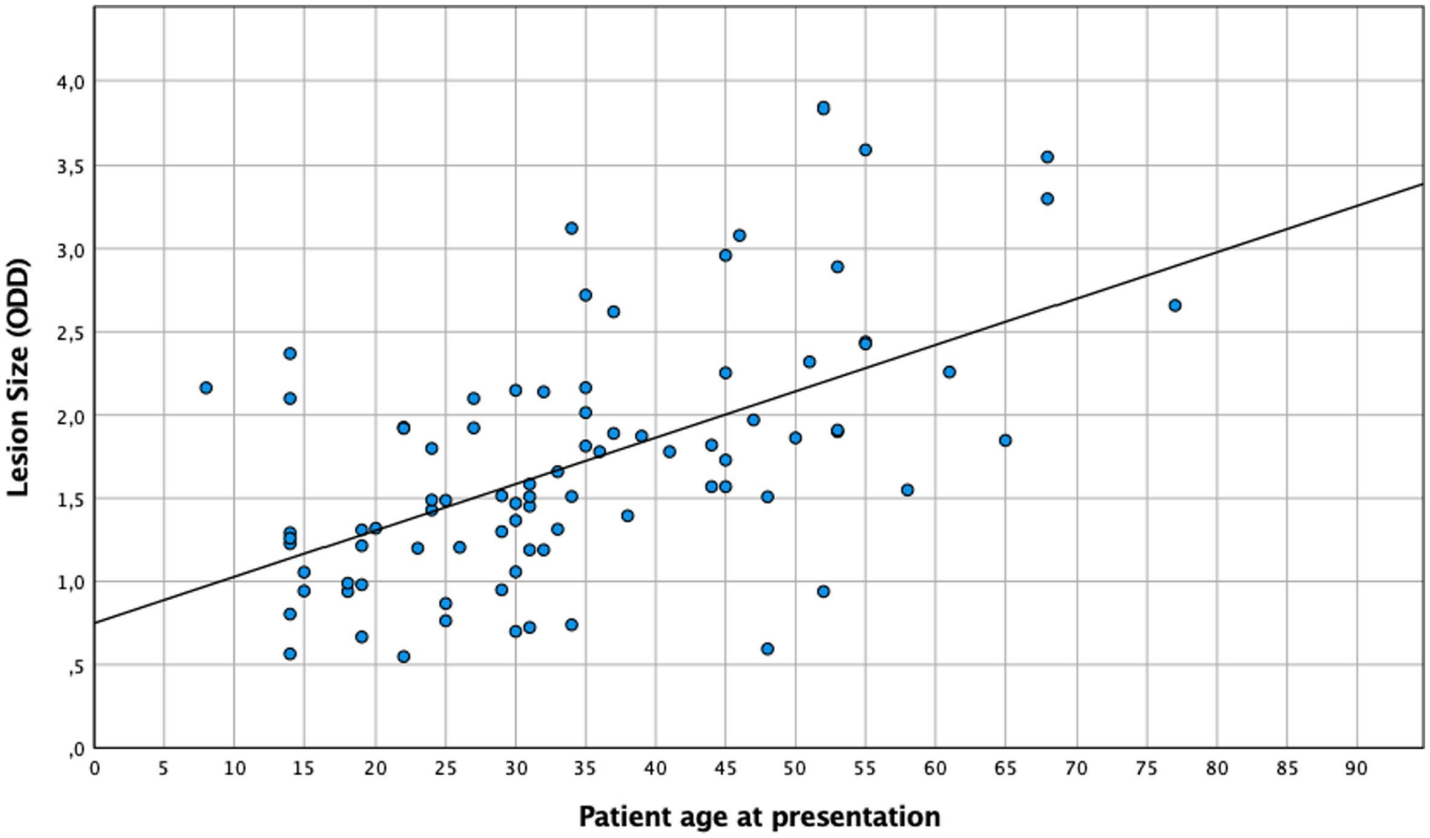
Lesion size in relation to patient age at presentation (*n* = 86). *R*^2^ = 0.32; *p* < 0.0005.

A central location of lesions was associated with younger age. Moreover, patient age had a significant effect on the severity of anterior and posterior inflammatory segment changes and lesion size (Table 2). When age and lesion size were analyzed as metric variables, we found similar results in the one-way ANOVA (*n* = 86, *p* < 0.005) and linear regression analysis (Figure 3, *R*^2^ = 0.32, *p* < 0.005). In the multivariate analysis (*n* = 204), an association between anterior chamber inflammation and patient age was not observed, while posterior segment inflammation and panuveitis were associated with older age. This association was confirmed by an ordinal logistic regression analysis (Table 2).

**Table 2:** Patient age and clinical presentation at baseline.

Characteristic	Number (*n*)	Age, mean in years (median) (SD)	*p*-value^u^	*p*-value^m^	Confidence interval	Odds ratio
**Manifestation mode**						
Primary OT	80	38.2 (32) (19.3)	0.4	0.4	0.99–1.04	1.01
Reactivated OT	195	36.3 (34) (15.5)	—	—	—	—
**Lesion location**						
Central (macula and peripapillary region)	135	34.0 (31) (14.9)	0.004	0.31	0.96–1.01	0.99
Peripheral	137	39.7 (36) (17.3)	—	—	—	—
**Lesion size**						
1 ODD	99	28.1 (25) (13.1)	<0.005	<0.005	1.05–1.09	1.07
2 ODD	74	38.4 (37) (14.6)	—	—	—	—
3 ODD	27	48.3 (46) (15.8)	—	—	—	—
4 ODD	4	52.8 (52) (5.2)	—	—	—	—
**Anterior chamber inflammation**						
Absent	154	34 (31.5) (15.8)	<0.005	0.28	0.99–1.04	1.02
Present	124	41.9 (41) (18)	—	—	—	—
**Vitreous inflammation**						
Absent	60	30.4 (29) (13.6)	<0.005	<0.005	1.02–1.09	1.06
Present	221	39.4 (36) (17.5)	—	—	—	—
**Anterior and posterior segment inflammation**						
Absent	166	33.8 (31) (15.6)	0.002	0.043	1–1.05	1.02
Present	112	43.1 (43.5) (18)	—	—	—	—
**Inflammatory activity**						
Absent in both compartments	47	30.2 (29) (13.9)	<0.005	<0.005	0.003–0.05	1.02
Anterior or posterior segment	119	35.2 (32) (16)	—	—	—	—
Anterior and posterior segment	112	43.1 (43.5) (18)	—	—	—	—
**Intraocular pressure**						
Normal (<22 mmHg)	212	38.4 (35.5) (16.9)	0.95	0.12	0.93–1.01	0.97
Elevated (>22 mmHg)	30	38.6 (36.5) (17.4)	—	—	—	—
**Complications**						
Absent	243	37.8 (35) (17.1)	0.66	0.63	0.98–1.03	1.01
Present	42	37.7 (34) (17.7)	—	—	—	—

Statistics: *t*-test, ^u^, univariate analysis; ^m^, multivariate analysis; ODD, optic disk diameter; SD, standard deviation. Bold values indicate significant findings.

### 3.4 Clinical presentation and lesion size

Beyond a total of 204 patients with defined lesion size, 99 (48.5%) of them had lesions of 1 ODD, 74 (36.3%) of 2 ODD, 27 (13.2%) of 3 ODD, and 4 (2%) of 4 ODD in size. Although this study was not powered to correlate the effect of immune state and lesion size, we also found that HIV-positive patients had larger lesions in both the univariate and multivariate analyses (*p* = 0.003).

### 3.5 Local antibody production, age, and lesion size

**Figure 4:**
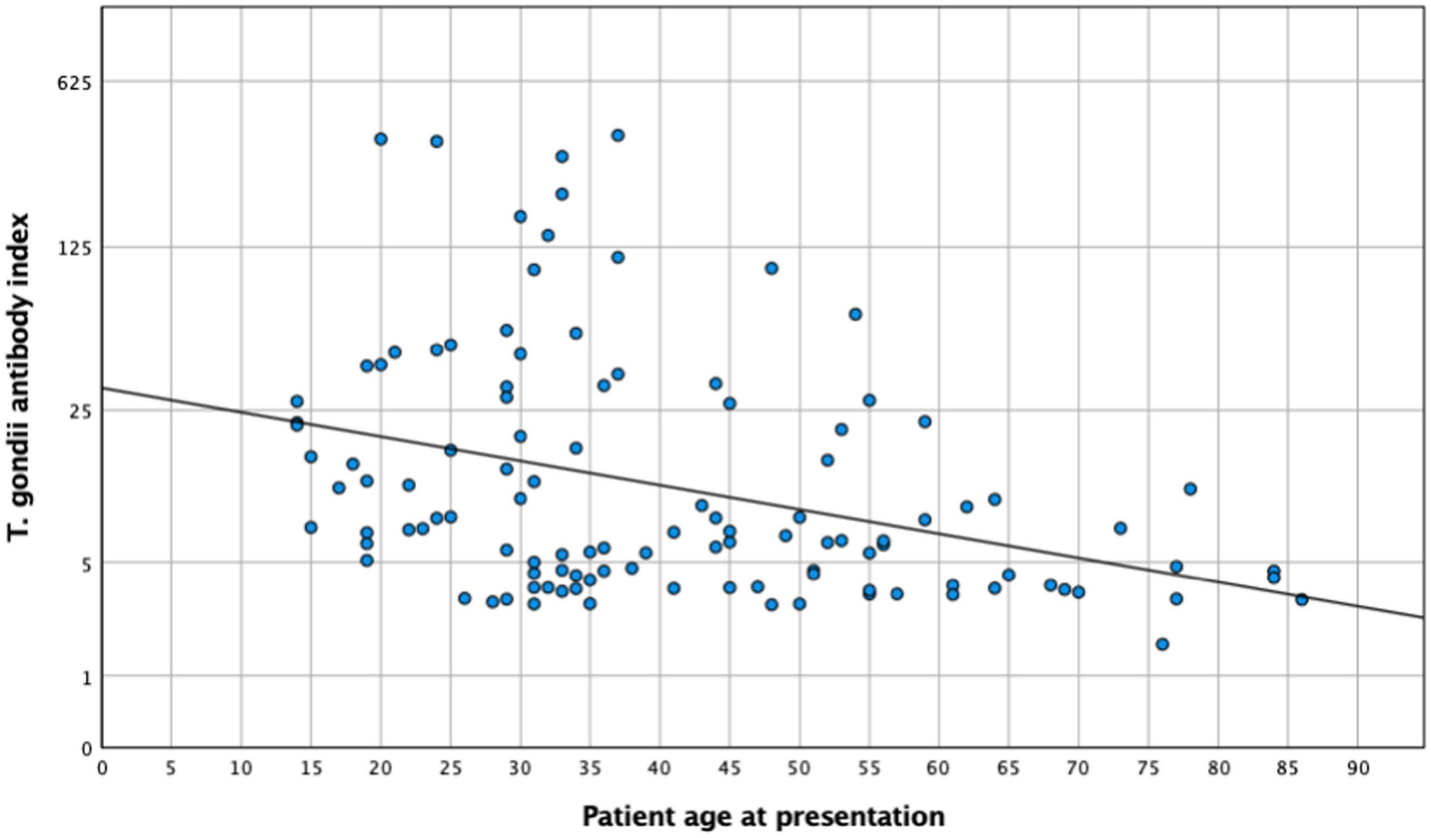
*Toxoplasma gondii* antibody index and patient age at presentation (*n* = 113). *R*^2^ = 0.045; *p* = 0.024.

An aqueous humor analysis was performed in 113 instances to confirm the clinical diagnosis with a mean antibody index (AI) of 31 ± 68.5 (median: 7; 1.71–369). Interestingly, younger patients presented a higher AI (mean < 35 years: 45.1 ± 82.7, median: 12.1; mean ≥35 years: 18.6 ± 50.5, median: 5.8; *t*-test: *p* = 0.046; Mann–Whitney *U*-test: *p* < 0.005). The metric analysis revealed a continuous decrease in the AI with increasing age (Figure 4, *p* = 0.024; *R*^2^ = 0.045), which was confirmed in the multivariate analysis (*p* = 0.036). Although no significant association between local antibody production and lesion size was observed in the univariate analysis (*n* = 64; *p* = 0.26) and a simple linear regression analysis (*p* = 0.5), the multiple linear regression analysis revealed a significant association between these two parameters (regression coefficient *B*: 38.6; *ß* = 0.4; *T* = 2.1; *p* = 0.043).

### 3.6 Complications

Initially, 35 patients (12.3%) presented with central retinal edema (macular or papillary) and 9 (3.2%) with retinal detachment. These complications were more common in patients with central lesions [22.4% (*n* = 30/134) vs. 6.0% (*n* = 8/133) of peripheral lesions; *p* < 0.005]. In the univariate analysis, we did not find a correlation between patient age and the occurrence of complications in this cohort. On the other hand, complications were associated with vitreous involvement (92.9% of patients with vitreous involvement compared to 76.9% of patients without it; *p* = 0.019) but not anterior chamber inflammation (52.4% of patients with anterior chamber inflammation compared to 42.3% of patients without it; *p* = 0.28). In the multivariate analysis, we found a strong correlation between central lesion location and complications (*n* = 267; *p* < 0.005), as well as between the presence of panuveitis and complications (*n* = 273; *p* = 0.035).

## 4 Discussion

The results of this cohort study indicate a strong and clinically relevant impact of age and inflammation on the presentation and course of toxoplasmic uveitis, which deserves further attention. Patients aged 35 years and above exhibited a lower baseline BCVA, with lesions larger and more frequently located in the periphery, while younger patients more often presented as central and bilateral disease (Figure 1). As reported previously (29), older patients in our series showed a more pronounced inflammatory response, particularly in the posterior segment. However, this is not specific; since the AI was more pronounced in younger individuals. Furthermore, this fact is also supported by a negative correlation between AI and age (Figure 4), which is an interesting finding that has not been reported previously. Finally, following the multivariate analysis, central lesions in younger individuals were more frequently associated with inflammatory complications.

These findings are well in line with previous observations from different European countries, indicating more severe disease and larger lesions in the immunocompetent elderly (30–32). Given the high prevalence of low-virulent type 2 strains in Europe, these findings cannot readily be explained by *T. gondii* virulence (12). Instead, this aspect may result from a less specific immune response against the parasite. This finding is new and is not so readily explained by the concept of immunosenescence, which would be accompanied by less severe inflammation. Our patients were with almost 38 years older than expected from previous studies (26–32 years) (29, 33–38), which may accentuate this finding. Presumably, this discrepancy is linked to the mainly urban population in our cohort. It is also well-conceivable that a referral bias with a more complicated clinical course of OT contributed to the older mean age of our patients.

Several factors may affect the functional outcome of OT, including patient age, the mode of OT (either primary OT or a recurrence), the location and size of a lesion, and secondary complications. Younger age has been reported to be associated with lower functional outcomes despite anti-parasitic therapy (39), though this finding has not consistently been supported (40).

Certainly, age is not necessarily directly linked to worse visual acuity. Various factors included in our multivariate analysis were, on the other hand, associated with older age and may have indirectly contributed to the outcome in our study population. Among these factors, size and location (29), the number of lesions (41, 42), and the severity of the posterior, but not the anterior, segment inflammation, have to be noted (29, 43–45). In addition, larger lesions, more severe inflammation, the occurrence of complications, and prolonged disease activity were more common in patients aged above 35 years (data not shown) (29).

Retinochoroiditis remains a rare (or frequently missed due to being asymptomatic) finding during primary infections and typically emerges months to many years thereafter (46–48). Consequently, primary OT will result in a vast majority of instances, from the reactivation of chronic toxoplasmosis, at least in Europe and North America with their low virulent strains. One hypothesis to explain our findings is that the risk of severe OT increases with age due to a declining specific immune function referred to as immunosenescence, suggesting that there would be a less severe immune response in the elderly; however, our patients aged above 35 years exhibited a stronger immune response compared to younger individuals.

Aging in general is associated with a slow decline and imbalance of physiological immune functions. This fact is indicated by an increasing, unspecific inflammatory state known as “inflammaging,” which occurs in response to a constant load of different self and foreign antigens. Inflammaging occurs in parallel with reduced specific immunity (49). Although aging and immunosenescence play well-defined roles in the immune response to and in the survival of other parasitic diseases such as Chagas disease, leishmaniosis, and malaria, their role in toxoplasmosis has yet to be established (21). Different Toxoplasma strains are associated with specific geographic regions, and the heterogenicity in virulence results in a differential severity of specific immune responses and the risk of recurrences (21). Archetypical type 2 old-world strains are known to be less virulent, which, in turn, may not only result in a higher survival rate in experimental Toxoplasma models but also result in a higher tissue load with parasite cysts (50). Adding thereto, the humoral immune response to low virulent strains is increasingly attenuated with increasing age (51).

From a clinical perspective on OT, our findings demonstrate an increased and longer lasting inflammatory response in patients above 35 years, which may be linked to the “inflammaging” phenomenon. We also found a decrease in specific antibody production at the local site, which is indicated by a lower AI, suggesting “immunosenescence” mechanisms (49). This decrease may in part be associated with an increased susceptibility of the elderly to infectious diseases and a decreased specific antigen response, as the patients in our cohort are older than previously reported (52).

Inflammaging and immunosenescence have received substantial attention in ocular disease in recent years, including age-related macular degeneration (53) and uveitis (54). While evidence on the humoral immune response with age is limited, an increasing cellular-immune dysfunction, a vanning proliferation of antigen-specific lymphocytes, a reduced cytokine production, and a lower activation level of cytotoxic and natural killer cells (55, 56) have been demonstrated. Adding thereto, an imbalance between memory and naive T cells, which reduces the response to new infectious antigens, has been observed (56–59). After the age of 50 years, there is also a decrease in regulatory T cells (Tregs), which might contribute to age-related phenomena such as increased inflammation and autoimmunity (55). Evidently, there is a continuous decline in the physiological immune response with increasing age, and the differences in our cohort, which was based on an arbitrarily set median age cutoff of 35 years, may indicate that differences do not strictly follow the biodynamics of age-related hormonal changes (60).

The correlation that we observed between lesion size and inflammatory intraocular findings with increasing age has previously been reported in studies from Europe (29) and South America. Dodds et al. reported more pronounced anterior and posterior segment inflammation in larger lesions and with increasing age (43). An increased vitreous haze in larger extramacular lesions was also consistent with our observations (43). It is possible that larger lesions result from a delayed or insufficient, specific immune response contributing to an increased parasite load and an increased, secondary inflammatory response in order to control parasite proliferation (43).

The strengths of this study include its large patient cohort, which was evaluated by experienced ophthalmologists using identical standards. It is important to note that a high proportion of cases are confirmed by aqueous humor analysis. In addition, the study covered a wide range of clinical aspects relevant to OT, allowing for a more comprehensive understanding of the impact of patient age on overall clinical presentation.

Beyond the limitations, our patient cohort may be prone to a selection bias of more severe cases due to the nature of our center. In addition, it was not always possible to accurately determine the onset of symptoms before the time of presentation, and some patients may have already been in the recovery phase while others were at the peak of the inflammatory phase, which may have resulted in some inaccuracies regarding the healing time. A notable limitation of our study lies in the absence of a comparable group of healthy individuals to differentiate a physiological decline in visual acuity from changes induced by OT. A decline in visual acuity with increasing age has been reported in healthy individuals starting from the age of 50 years, but namely above 70 years of age, after having reached full development around school age (61, 62). Since the age discriminator of 35 years in our series lays considerably below the expected age for a physiological visual decline, we acknowledge a potential, but probably irrelevant, contribution of age to the observed findings.

Lesion size, especially in cases of high myopia or vitreous body opacity, may be associated with lowered accuracy; however, the large sample size and a presumed similar distribution of these factors between both groups likely balanced our results. Our observations were not carried forward to replace missing data, given the sample size in this large, retrospective cohort study over a period of more than 20 years. It seems nevertheless unlikely that there is a relevant impact of missing data on the outcomes reported here. Finally, it must be kept in mind that, in a retrospective setting, causal relationships cannot be established. Although the risk of type I error cannot be ruled out, the correlations found in our cohort were largely consistent with previous studies and were in line with pathogenetic considerations.

Thus, it seems that patients with primary OT have larger lesions and are older than patients with recurrent disease (29), but they also have lower local antibody production (63). This aspect supports the robustness of our findings and is well-explained by evidence gained from animal experiments (64).

In conclusion, in a large human cohort with OT, our data show that, while the inflammatory response in general increases with age, the specific local humoral immune response declines. As a result, we found larger lesions located predominantly in the periphery and a longer resolution time for inflammatory changes with increasing age. Taken together, our findings support the concept of immunosenescence and inflammaging at the level of ocular disease.

## Data availability statement

The raw data supporting the conclusions of this article will be made available by the authors, without undue reservation.

## Ethics statement

The studies involving humans were approved by Medical Ethics Committee, Charité Universitätsmedizin Berlin (EA4/034/16). The studies were conducted in accordance with the local legislation and institutional requirements. The participants provided their written informed consent to participate in this study.

## Author contributions

AE: Data curation, Formal analysis, Investigation, Methodology, Visualization, Writing – original draft. JG: Funding acquisition, Supervision, Writing – review & editing. UP: Conceptualization, Data curation, Funding acquisition, Investigation, Supervision, Validation, Writing – review & editing.
